# Supplementary material for: Dynamic HIV-1 spike motion creates vulnerability for its membrane-bound tripod to antibody attack
Source: Nat Commun. 2022 Oct 27;13:6393. doi: 10.1038/s41467-022-34008-y (PMC9610346; doi:10.1038/s41467-022-34008-y)
Supplement: Supplementary file 1 — Supplementary Information [file 41467_2022_34008_MOESM1_ESM.pdf]

Supplementary Materials for

Dynamic HIV-1 spike motion creates vulnerability for membrane-bound tripod to antibody attack

Shuang Yang[1†], Giorgos Hiotis[1,2†], Yi Wang[3,4], Junjian Chen[3,4], Jia-huai Wang[3,5-7], Mikyung Kim[3,8], Ellis L. Reinherz[3,4*] and Thomas Walz[1*]

[1]Laboratory of Molecular Electron Microscopy, The Rockefeller University; New York, NY, USA.

[2]Tri-Institutional PhD Program in Chemical Biology, The Rockefeller University; New York, NY, USA.

[3]Laboratory of Immunobiology, Department of Medical Oncology, Dana-Farber Cancer Institute; Boston, MA, USA.

[4]Department of Medicine, Harvard Medical School; Boston, MA, USA.

[5]Department of Biological Chemistry and Molecular Pharmacology, Harvard Medical School; Boston, MA, USA.

[6]Department of Cancer Biology, Dana-Farber Cancer Institute; Boston, MA, USA.

[7]Department of Pediatrics, Harvard Medical School; Boston, MA, USA.

[8]Department of Dermatology, Harvard Medical School; Boston, MA, USA.

† These authors contributed equally to this work.

* Corresponding authors: Emails: ellis_reinherz@dfci.harvard.edu ; twalz@rockefeller.edu

Structure of the Env protein with three bound 4E10 Fabs

The three 4E10 Fabs in our structure bind to the ectodomain quite differently than in the previous structures of the ectodomain in complex with three Fabs of bnAbs VRC42.01 and 10E8 (Supplementary Fig. 7c).[1] The 4E10 Fabs use binding mode 2, in which the Fabs extend into the space that would normally be occupied by the membrane, which should not occur *in situ*. In contrast, in the structures of the ectodomain in complex with three VRC42.01 and 10E8 Fabs, those extend away from the membrane in the same direction as the ectodomain.

The 4E10 Fabs bind in three respects differently from each other, introducing asymmetry in the gp145•3Fab complex. First, the three Fabs are not evenly distributed around the ectodomain trimer. Rather than being separated by equal 120º angles, when viewed in the direction from the membrane to the ectodomain, the angles from Fab1 to Fab2 to Fab3 are 96º, 114º and 150º (Fig. 3e). Similarly asymmetric distributions of MPER Fabs were also observed in the previously determined Env structures in complex with three VRC42.01 and 10E8 Fabs (Supplementary Fig. 7c). Second, the angle of the 4E10 Fab with respect to the three-fold axis of the ectodomain are 150º, 140º and 140º for Fabs 1, 2 and 3, respectively. Third, the plane of Fab1 and Fab2 is perpendicular to the membrane plane, while the plane of Fab3 is rotated by ~30º with respect to the other two Fabs. As the result of Fab1 and Fab2 being much closer together, we find that Fab1 utilizes its CDRH3 loop to also interact with the MPER-N segment from the adjacent gp145 protomer to which Fab2 is bound (Supplementary Fig. 7d). While Fab1 forms the interaction with its MPER epitope seen in the crystal structure, its Trp100 residue also forms aromatic stacking and hydrophobic interactions with Trp666, Leu669 and Trp670 of the neighboring MPER-N segment (Supplementary Fig. 7d), which is not observed for the other two Fabs.

SUPPLEMENTARY FIGURES

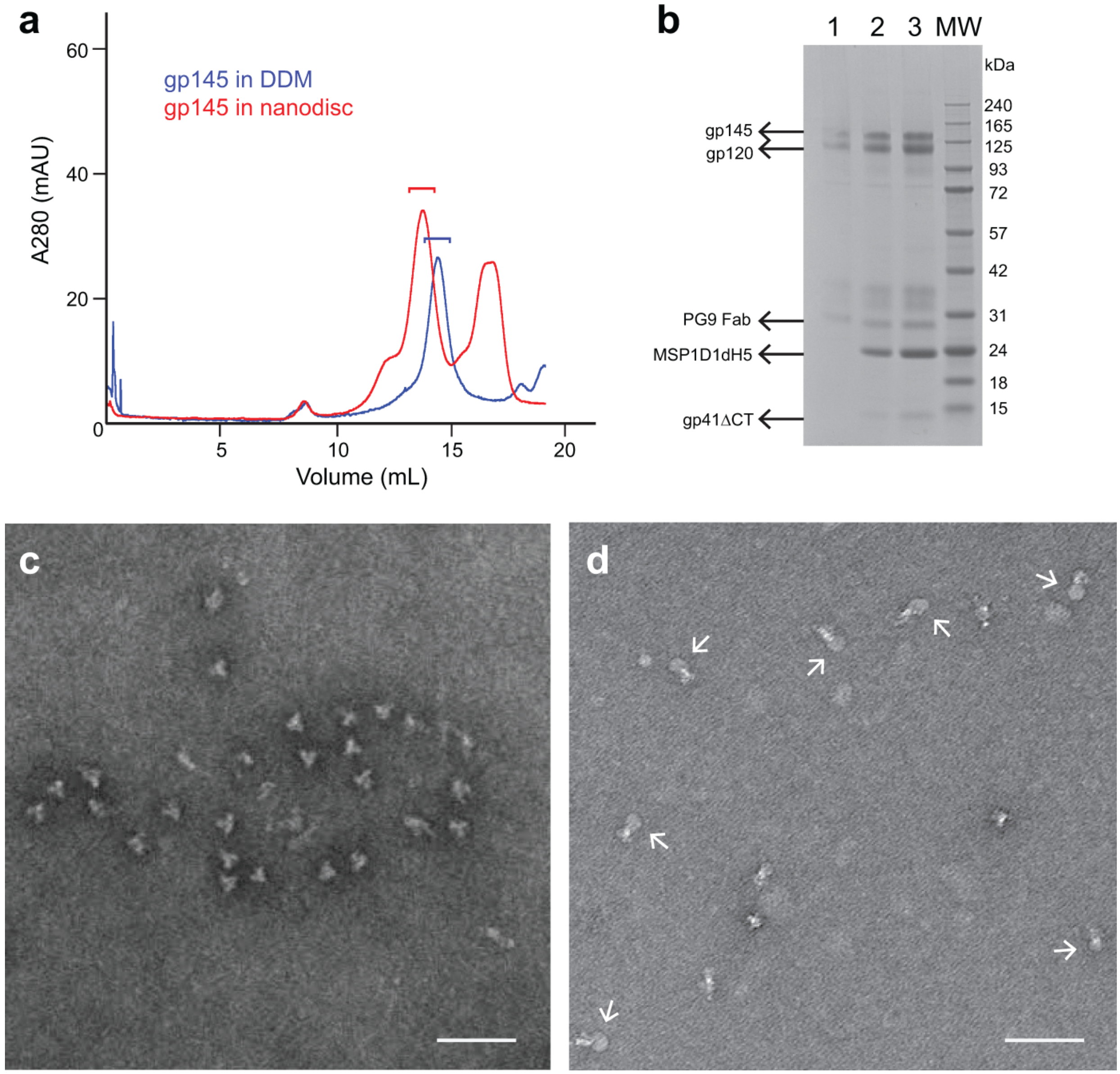

Supplementary Figure 1. Preparation of nanodisc-embedded gp145 in complex with 4E10 Fabs. (**a**) SEC elution profiles showing a single peak for gp145 in DDM (blue line) and two peaks for gp145 in nanodiscs (red line). The peak at a lower elution volume than the peak in the DDM sample represents gp145-containing nanodiscs, whereas the peak at a higher elution volume than the peak in the DDM sample represents empty nanodiscs. Brackets indicate the fractions that were used for negative-stain EM imaging. (**b**) Coomassie Brilliant Blue-stained reducing (5% β-mercaptoethanol) SDS-PAGE gel of gp145 in DDM (lane 1) and in nanodiscs (lanes 2 and 3; sample in lane 2 is two-fold dilution of sample in lane 3). (**c** and **d**) Negative-stain EM images of gp145 in DDM (**c**) and nanodiscs (**d**). Images are representative of ~10 images taken from each sample. Arrows in panel (**d**) point to nanodiscs. Scale bars: 50 nm.

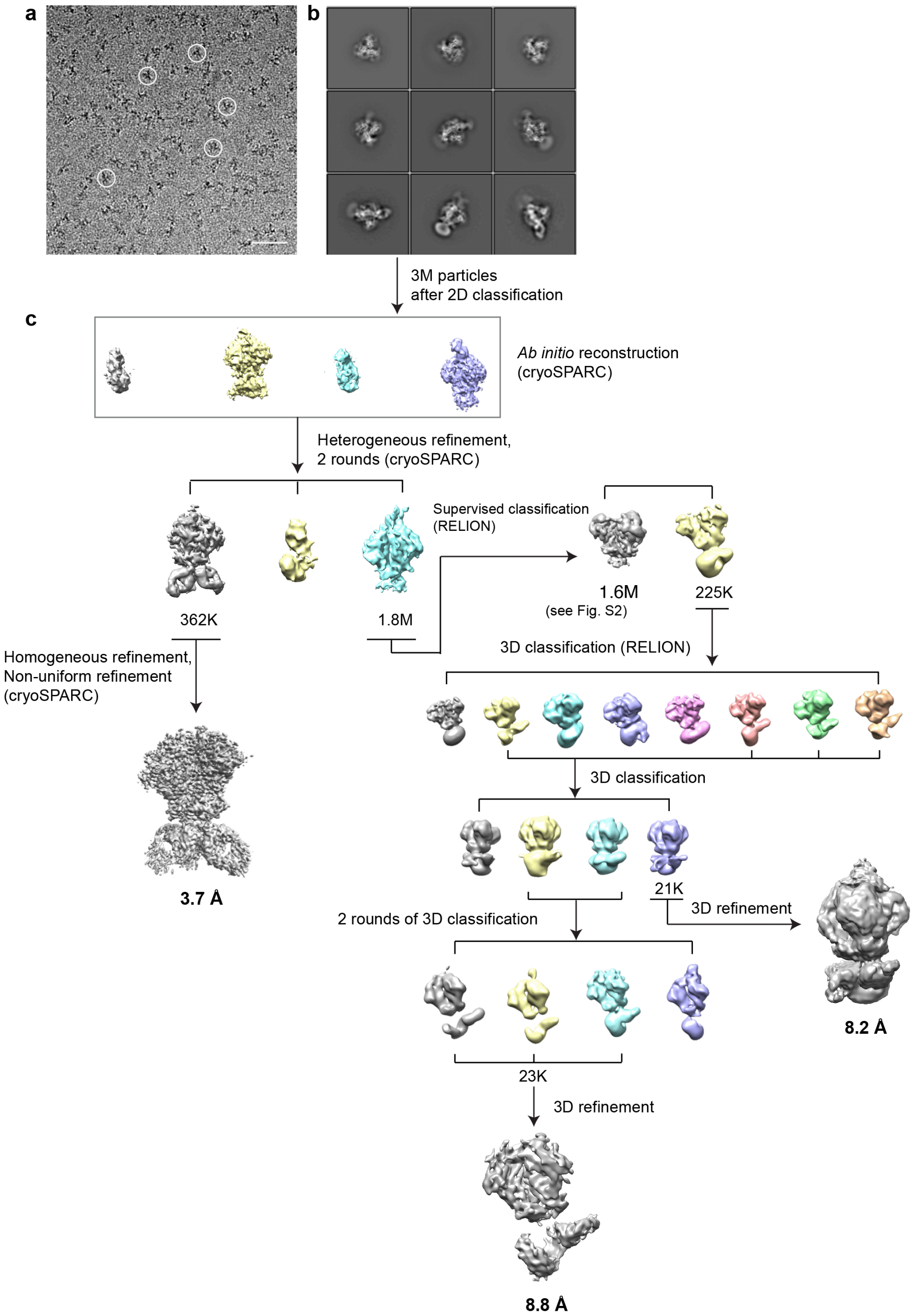

Supplementary Figure 2. Cryo-EM image processing of nanodisc-embedded gp145 incubated with 4E10 Fabs. (**a**) Representative cryo-EM image of vitrified sample selected from a dataset of 30,404 images. Some particles are circled. Scale bar: 50 nm. (**b**) Selected 2D-class averages obtained with RELION-3. Side length of individual averages: 39.6 nm. (**c**) Image-processing workflow for 3D classification and refinement in cryoSPARC and RELION-3 that resulted in density maps of the gp145•3Fab complex at 3.7-Å resolution, the gp145•2Fab complex at 8.2-Å resolution, and the gp145•1Fab complex at 8.8-Å resolution. See Methods and Supplementary Table 1 for details.

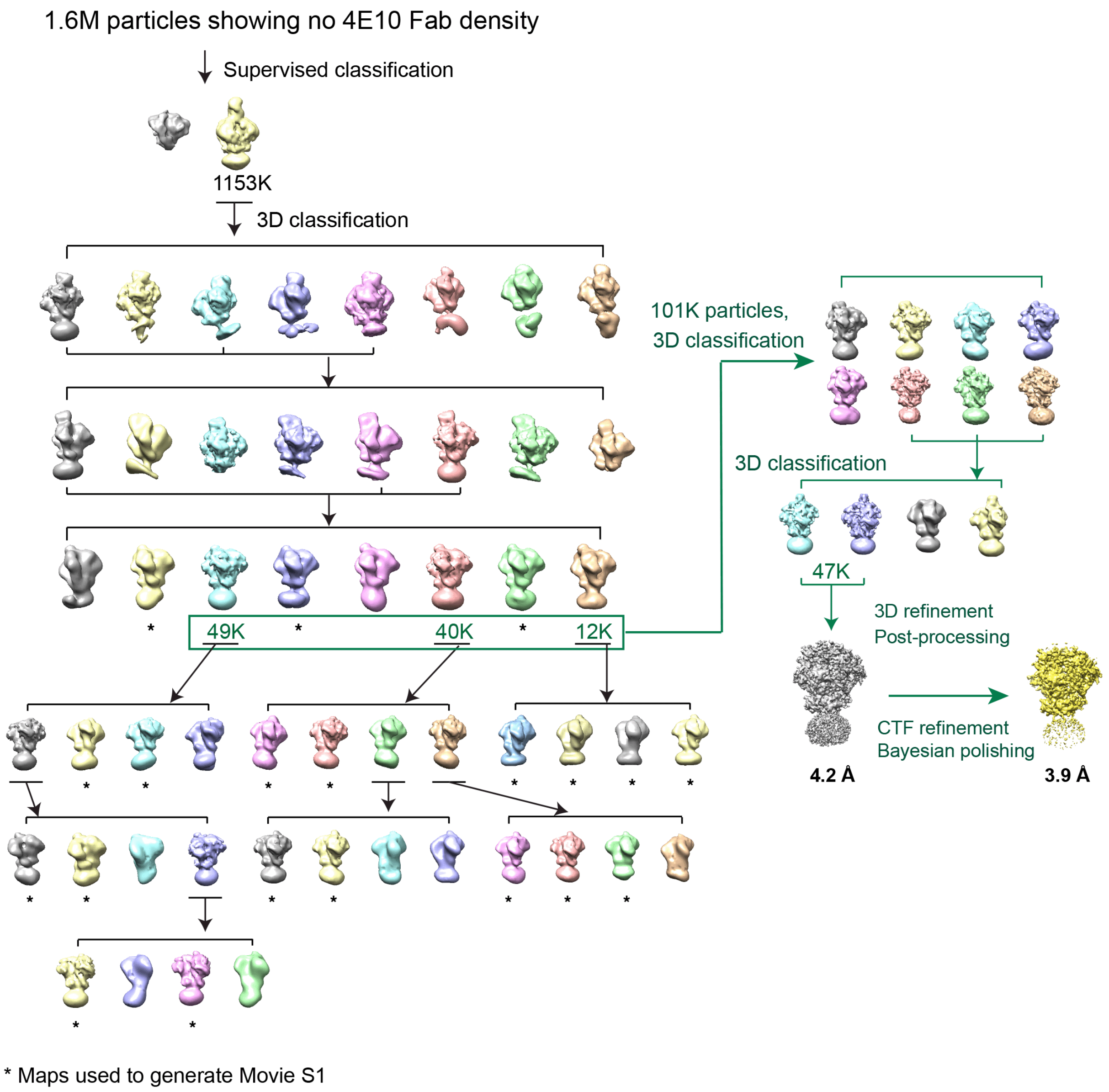

Supplementary Figure 3. Cryo-EM image processing of nanodisc-embedded gp145 incubated with 4E10 Fabs. Continued from Supplementary Fig. 2. Image-processing workflow for 3D classification and refinement in RELION-3 that resulted in a density map of nanodisc-embedded gp145 without bound Fab at 3.9-Å resolution. Maps labelled with * were used to generate Supplementary Movie 1. See Methods and Supplementary Table 1 for details.

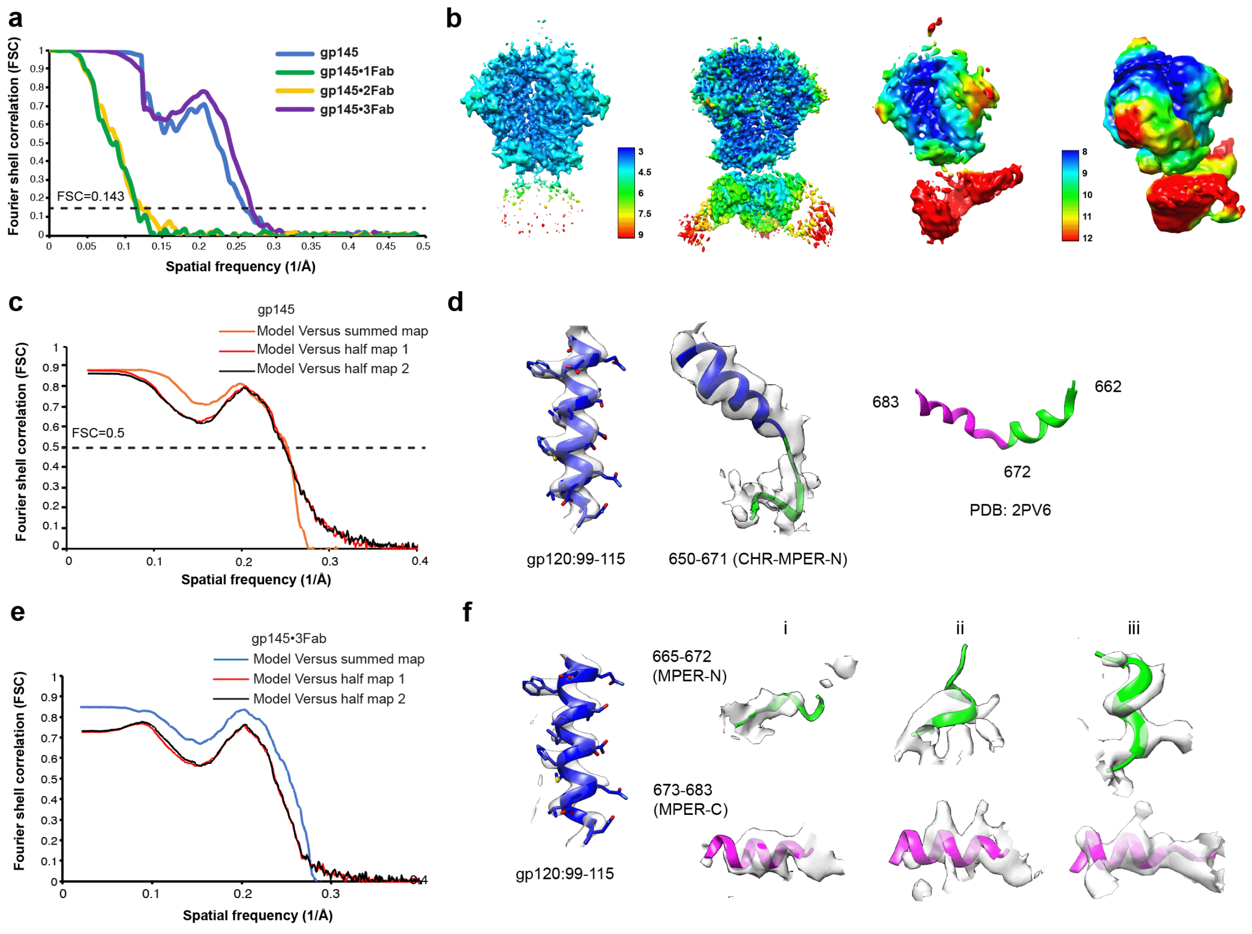

Supplementary Figure 4. FSC curves, local-resolution maps and local densities. (**a**) FSC curves calculated between independently refined half maps for gp145 map by itself (blue), the gp145•1Fab complex (green), the gp145•2Fab complex (yellow), and the gp145•3Fab complex (purple). (**b**) From left to right: local-resolution maps as determined by the ResMap algorithm implemented in RELION-3 for gp145 by itself and the gp145•3Fab complex with the resolution scale shown in between, and local-resolution maps for the gp145•1Fab complex and the gp145•2Fab complex with the resolution scale shown in between. (**c**) Cross-validation FSC curves for nanodisc-embedded gp145 by itself: red, refined model *versus* half map 1 used for refinement (work map); black, refined model *versus* half map 2 not used for refinement (free map); orange, refined model *versus* the combined final map. The similarity of the 'work' and 'free' curves suggests no substantial over-fitting. (**d**) Selected local cryo-EM densities for the map of gp145 by itself (left) and the NMR structure of the MPER used for docking of the N-segment into the cryo-EM density. (**e**) Cross-validation FSC curves for nanodisc-embedded gp145•3Fab complex: red, refined model *versus* half map 1 used for refinement (work map); black, refined model *versus* half map 2 not used for refinement (free map); orange, refined model *versus* the combined final map. The similarity of the 'work' and 'free' curves suggests no substantial over-fitting. (**f**) Selected local cryo-EM densities for the map of the gp145•3Fab complex. The densities representing the MPER-N and MPER-C segments bound by Fab1 (i), Fab2 (ii) and Fab3 (iii) are shown.

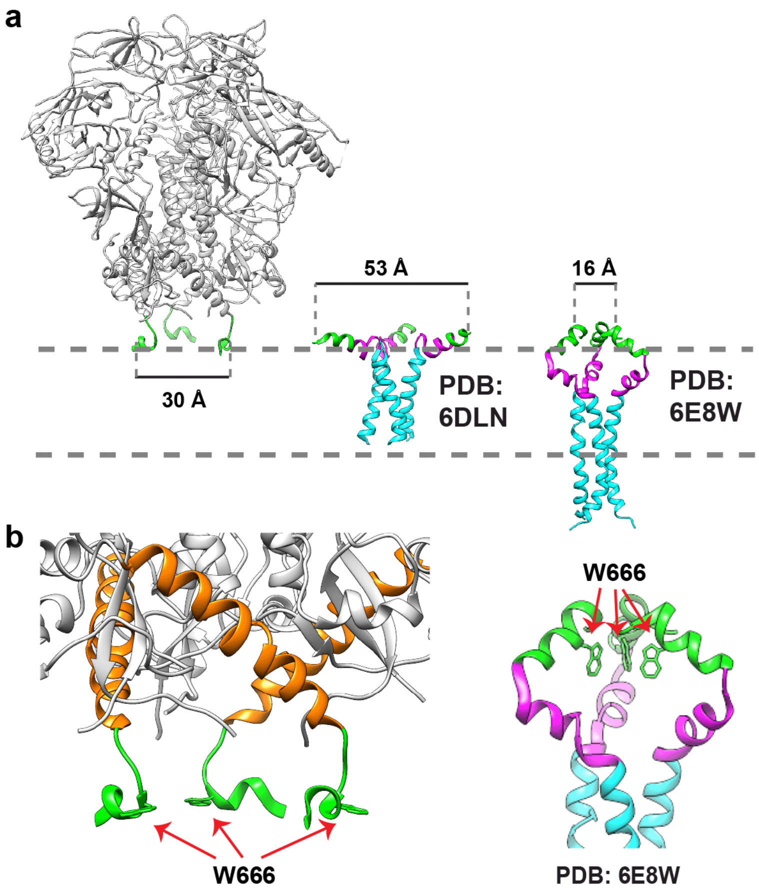

Supplementary Figure 5. Comparison of the MPER segments in the cryo-EM structure of nanodisc-embedded gp145 with those in NMR structures. The gp145 ectodomain is shown in gray, CHR in orange and MPER-N in green, MPER-C in magenta, and the transmembrane domain in blue. (**a**) The distance between the N-terminal ends of the MPER segments, measured between the Cα atoms of residue Lys665 from adjacent protomers, in the cryo-EM structure (this study) and two previously determined NMR structures (PDB: 6DLN and PDB: 6E8W).[2,3] Dashed grey lines indicate the membrane. (**b**) Comparison of the dispersed MPER-N segments in the cryo-EM structure (this study; left panel) with the converged MPER-N segments in one of the NMR structures (PDB: 6E8W; right panel).[3] The side chains of residue Trp666 are shown in stick representation.

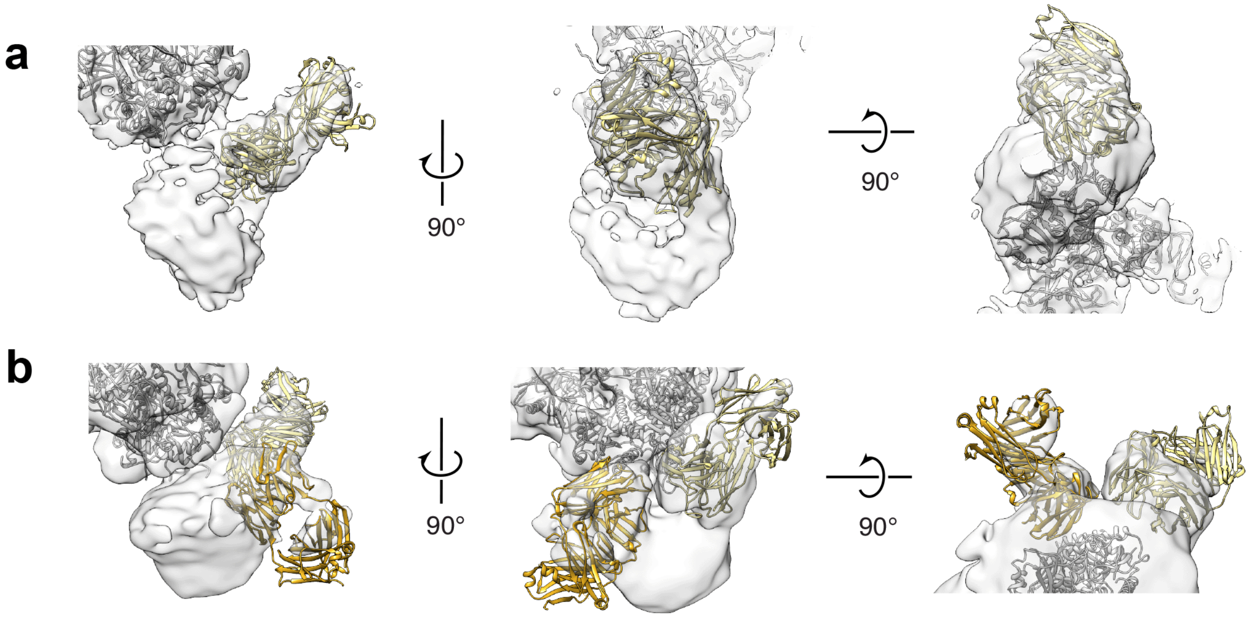

Supplementary Figure 6. Docking of the 4E10 Fab crystal structure into the maps of nanodisc-embedded gp145 in complex with one Fab (gp145•1Fab) and two Fabs (gp145•2Fab). (**a** and **b**) Three orthogonal views that show the docking of the 4E10 Fab crystal structure (PDB: 4XC3)[4] into the gp145•1Fab map (**a**) and the gp145•2Fab map (**b**). The gp145 ectodomain is shown in gray and the two Fabs in yellow and gold.

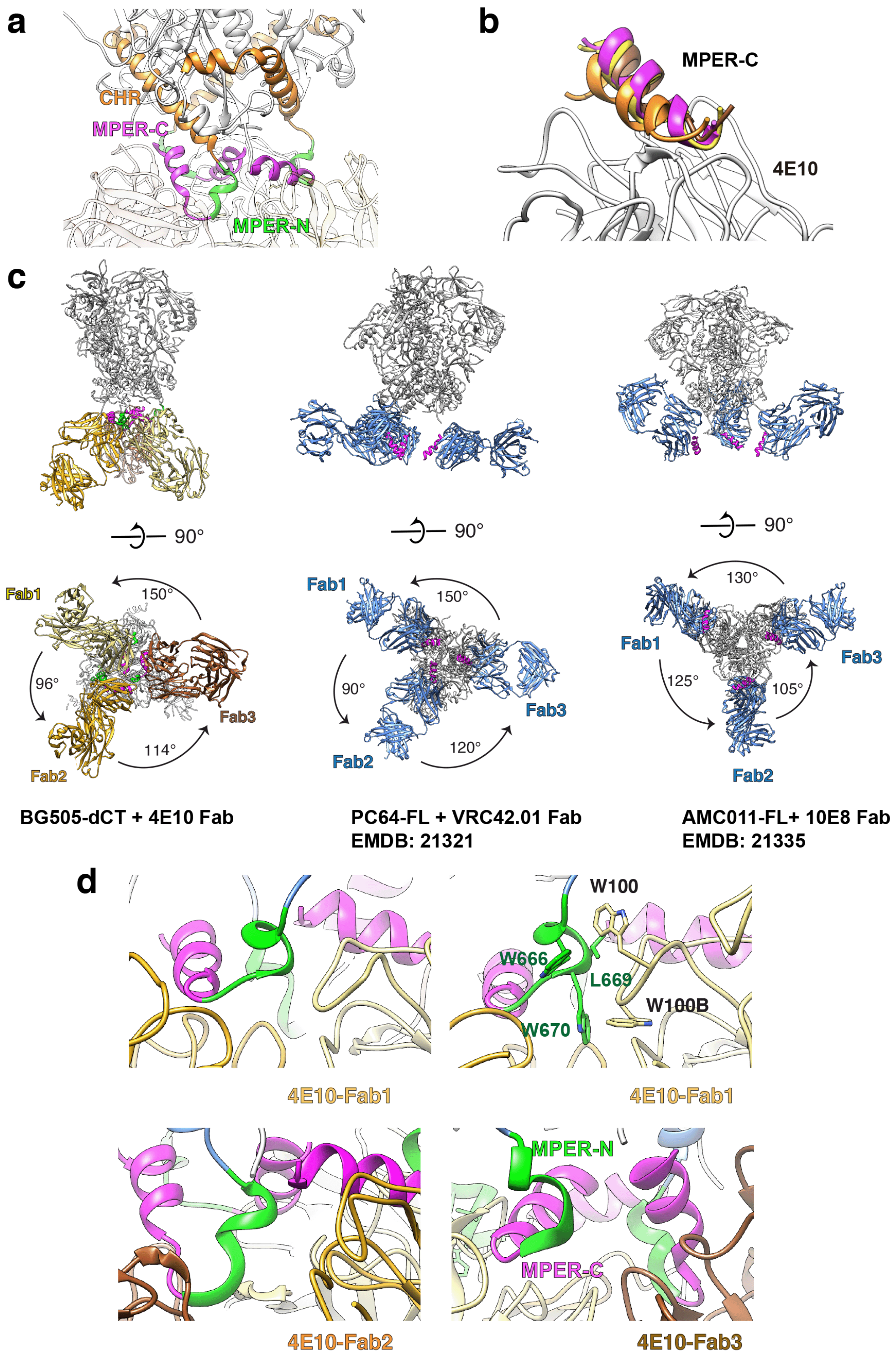

Supplementary Figure 7. Connection of the MPERs to the ectodomain and their interaction with bnAbs. (**a**) Close-up view of the connections of the MPERs (MPER-N in green and MPER-C in magenta) with the CHRs (orange) of the ectodomain in the gp145•3Fab structure. (**b**) Interaction of the three MPER-C segments in the gp145•3Fab structure (yellow, gold and brown) and the MPER-C epitope in the crystal structure (magenta) with the 4E10 Fab. The structures

were overlaid based on the Fab structure. (**c**) Cryo-EM structures of HIV-1 Env in complex with three anti-MPER bnAb Fabs (colored in yellow, gold and brown) seen parallel (top) and perpendicular to the membrane plane (bottom). Left panels: BG505-dCT gp145 with three 4E10 Fabs (this study). All three Fabs are bound in mode 2 and extend into the space that would normally be occupied by the membrane. Middle panels: Map: PC64 gp160 with three VRC42.01 Fabs (EMDB:21321)[1]; the coordinates used for docking are 5I8H for the gp160 ectodomain[5] and 6MTP for the VRC42.04 Fab (blue) in complex with the gp41 peptide (magenta).[6] Right panels: AMC011 gp160 with three 10E8 Fabs (PDB: 6PVX; colored in blue).[1] The Fabs seen in the two previous structures (middle and right panels) are bound to Env in mode 1 and extend away from the membrane. The angles between the three Fabs differ in the three structures. (**d**) The region between the 4E10 Fabs and the MPER-N segments from the neighboring protomers. Fab1 is closer to the neighboring MPER-N segment, possibly forming hydrophobic interactions. The residues involved in these putative interactions are shown in stick representation. Fabs 2 and 3 are further away from their neighboring MPER-N segments and are thus unlikely to from hydrophobic interactions. The Fabs are shown in yellow, gold and brown, and the green and magenta colors are used to delineate the MPER-N and MPER-C segments, respectively.

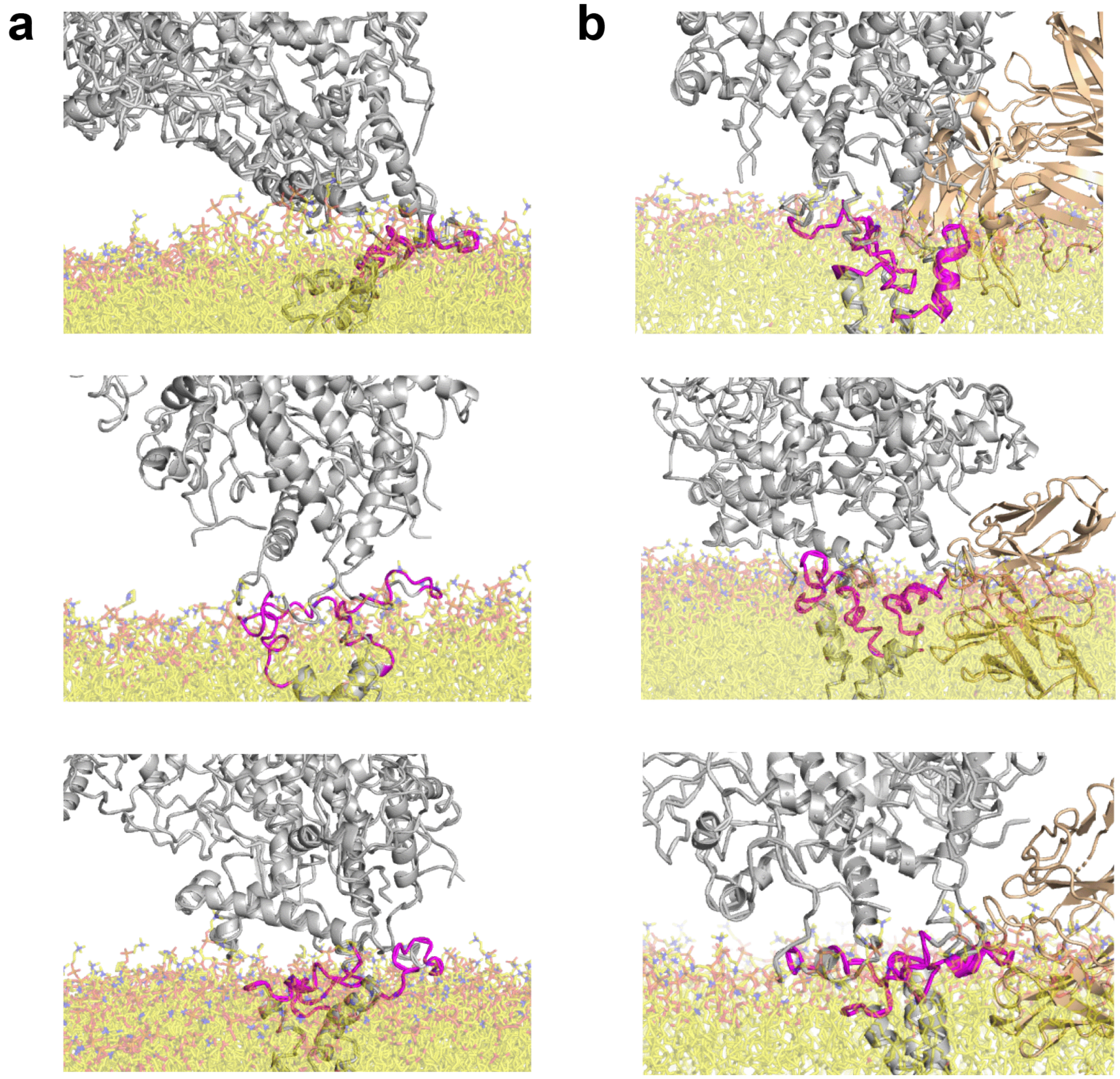

Supplementary Figure 8. All-atom views generated from snapshots of the coarse-grained molecular-dynamics simulations of membrane-embedded gp145 showing the ectodomain at high tilts. (**a**) Three snapshots in which the MPER-C segment lost its α-helical secondary structure, thus preventing reliable docking of the 4E10 Fab. (**b**) Three snapshots in which the MPER-C segment remained α-helical, but where docking of the 4E10 Fab resulted in steric clashes with the ectodomain or the Fab being partially buried in the lipid bilayer. The gp145 ectodomain is shown in gray, the MPER-C in magenta, the bound Fab in gold, and the lipid bilayer in yellow.

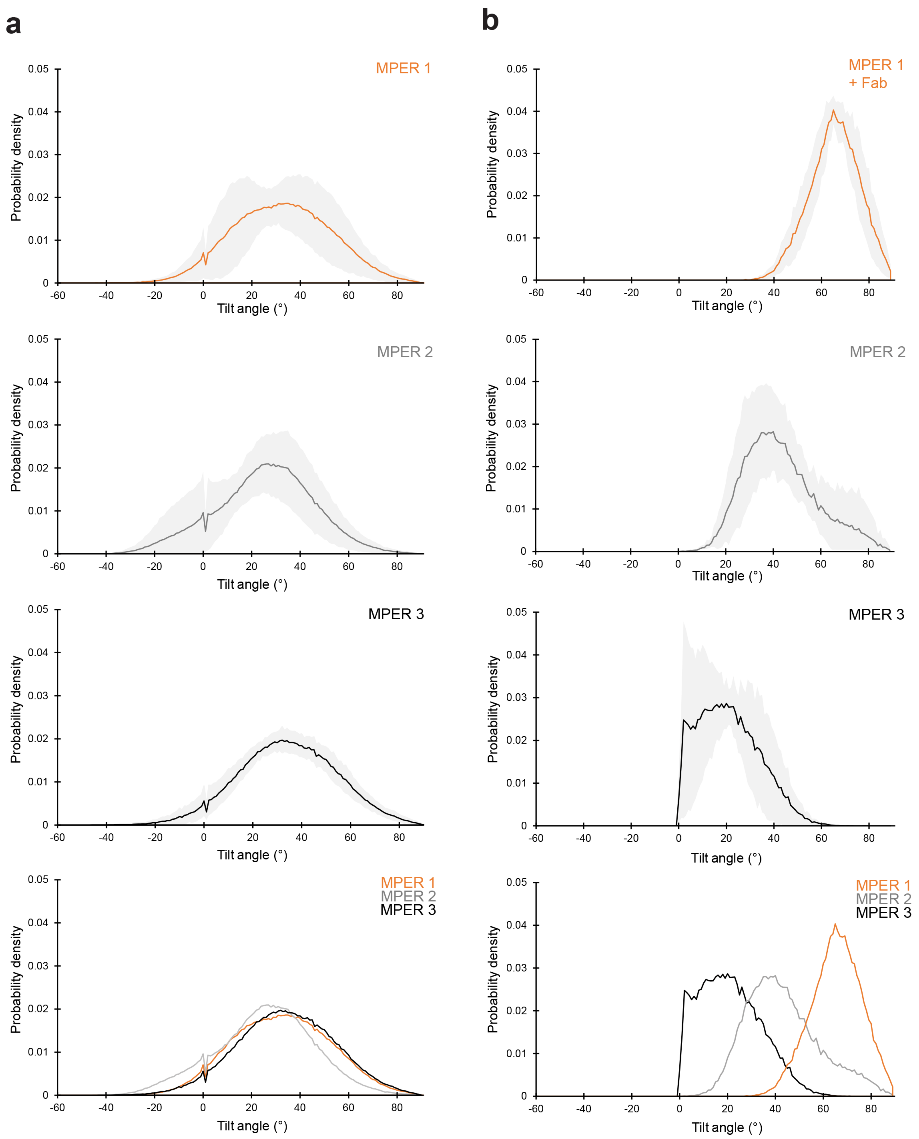

Supplementary Figure 9. Distribution of the angles adopted by the three MPER-C segments in coarse-grained molecular-dynamics simulations of membrane-embedded gp145. (**a**) Graphs showing the distribution of the angles adopted by the MPER-C segments in unliganded gp145, summarizing data from all five repeats. The top three panels show the angle distribution for the three MPER-C segments individually, with the solid lines representing the mean and the grey bands indicating the standard deviation. The bottom panel shows an overlay of the angle distribution for the three MPER-C segments, demonstrating that the three MPER-C segments in unliganded gp145 show comparable behavior. (**b**) Graphs showing the distribution of the angles adopted by the MPER-C segments in 4E10 Fab-bound gp145, summarizing data from all five repeats. The top three panels show the angle distribution for the three MPER-C segments individually, with the solid lines representing the mean and the grey bands indicating the standard deviation. The bottom panel shows an overlay of the angle distribution for the three MPER-C segments, demonstrating that the three MPER-C segments in 4E10 Fab-bound gp145 adopt distinct angles.

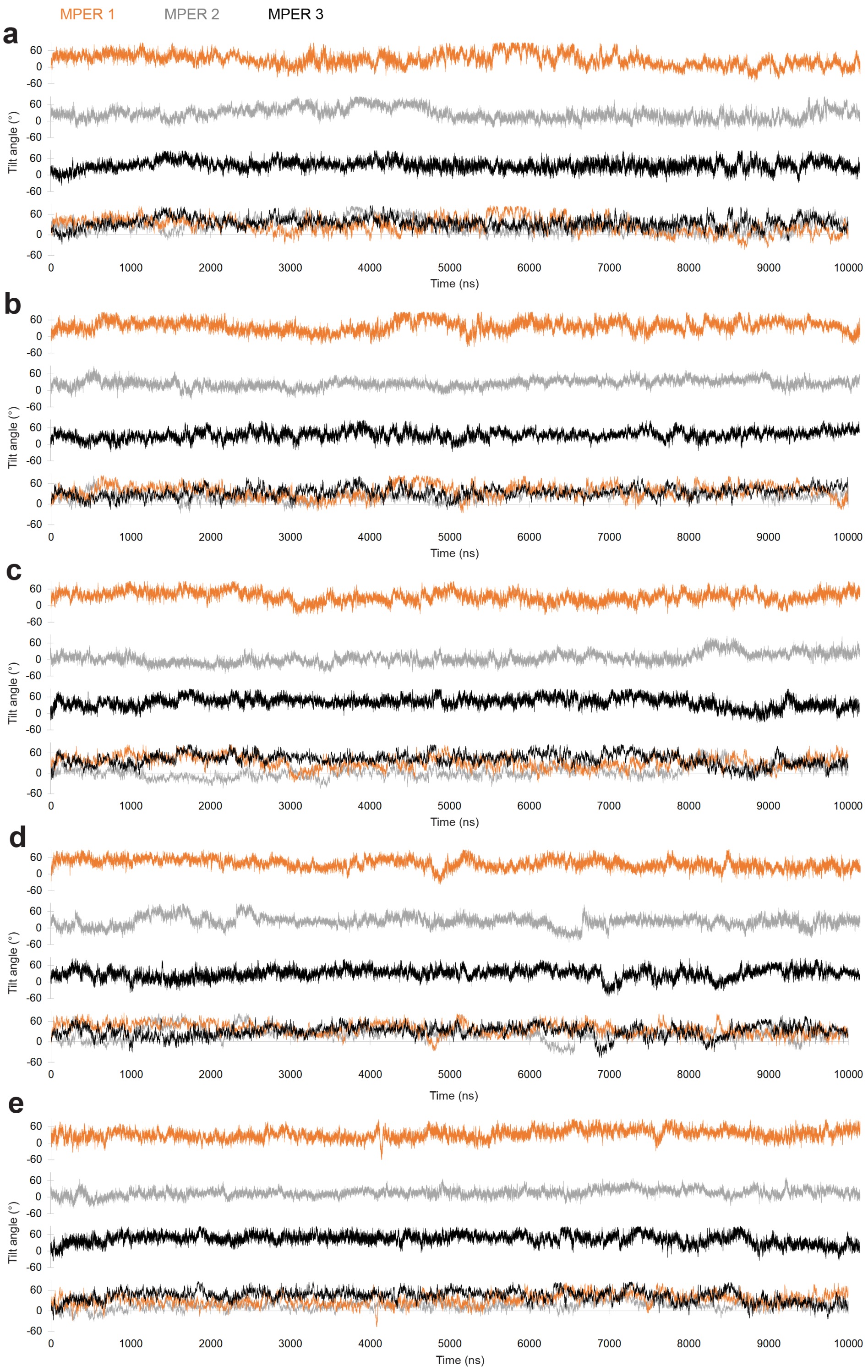

Supplementary Figure 10. Angles of the three MPER-C segments adopted over time in coarse-grained molecular-dynamics simulations of unliganded gp145. (**a-e**) For each of the five repeats, the angles adopted by each MPER-C segment is shown on their own (top three panels) as well as overlaid (bottom panel).

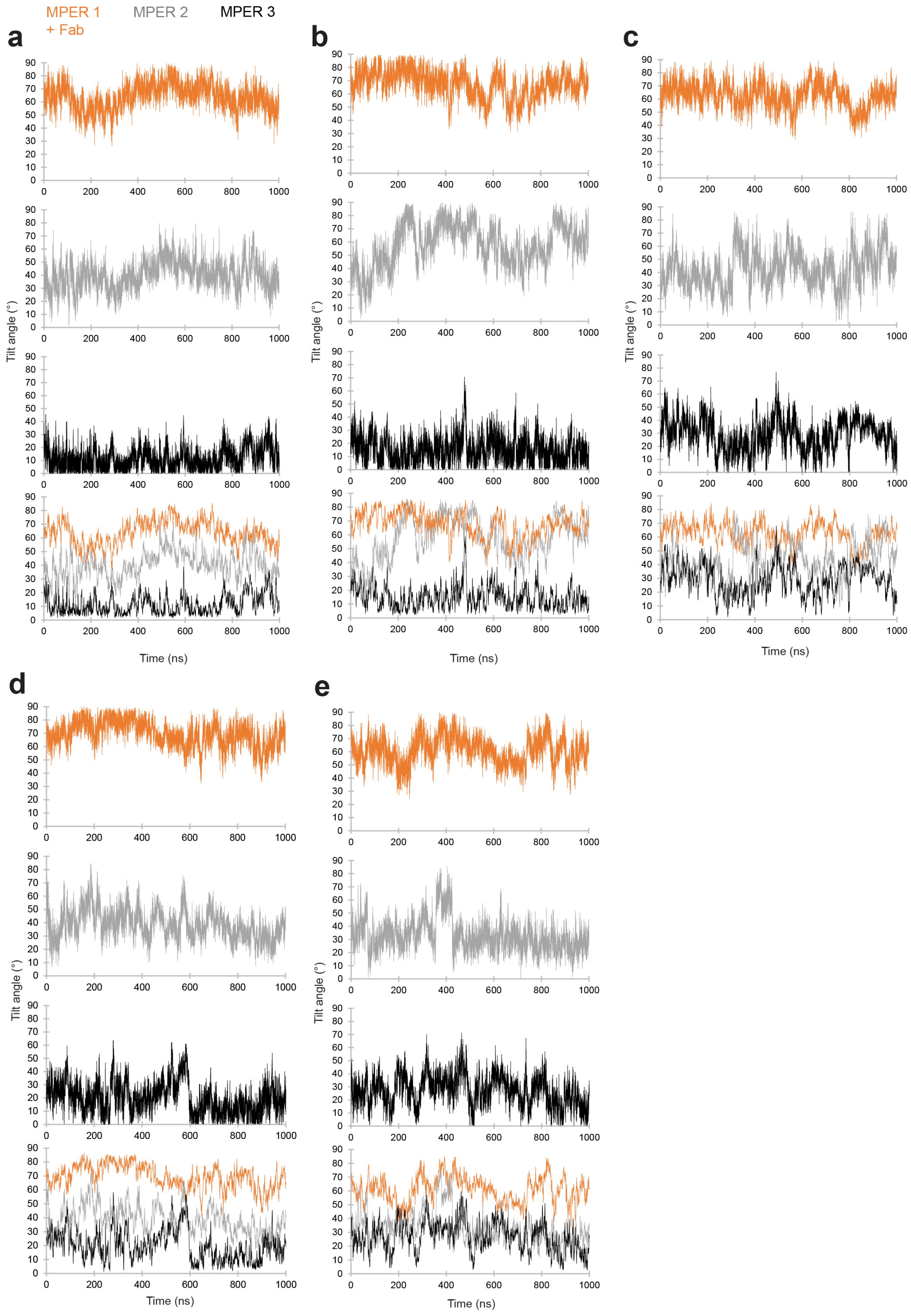

Supplementary Figure 11. Angles of the three MPER-C segments adopted over time in coarse-grained molecular-dynamics simulations of 4E10 Fab-bound gp145. (**a-e**) For each of the five repeats, the angles adopted by each MPER-C segment is shown on their own (top three panels) as well as overlaid (bottom panel).

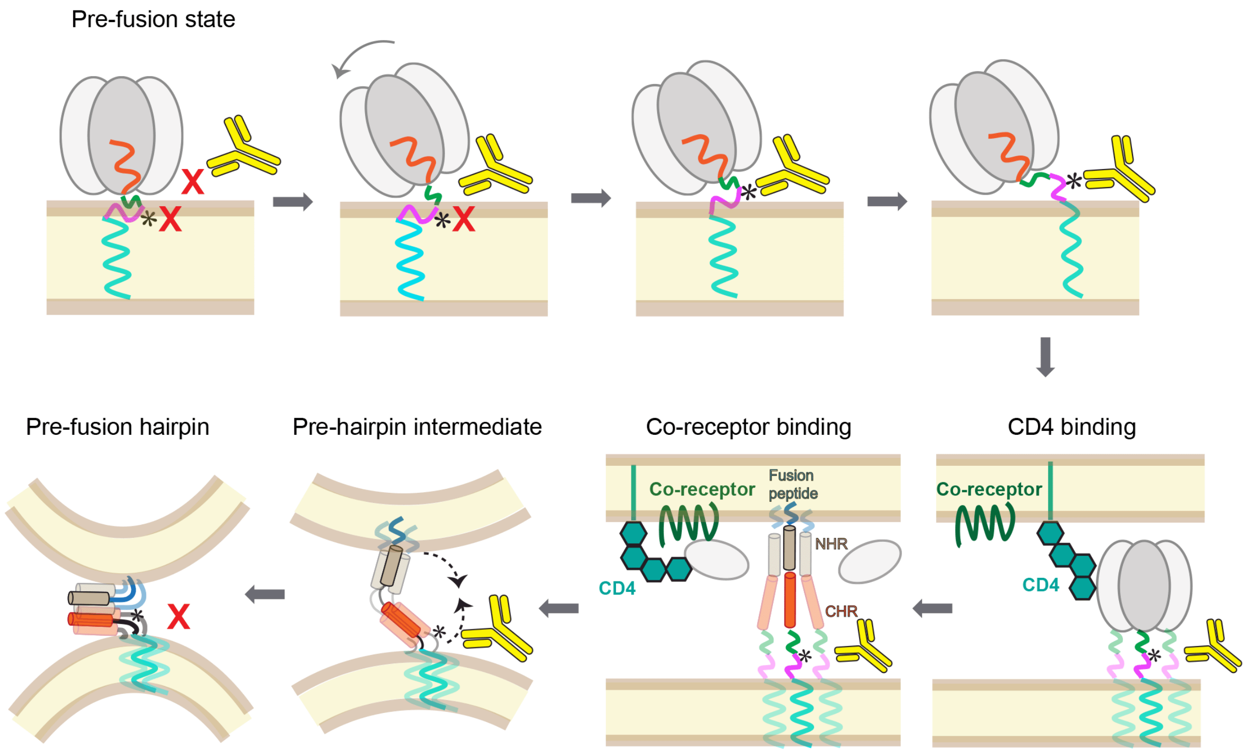

Supplementary Figure 12. The stepwise binding of the anti-MPER bnAb 4E10 to the Env protein and exposure of MPER epitope during different states of the fusion process. Upper panels, see Figure 6a. Lower panels: While the current study establishes that bnAb 4E10 (and possibly other anti-MPER bnAbs) can bind to HIV-1 Env in the prefusion state, the epitope (*) may also be accessible after CD4 engagement, gp120 shedding and during the pre-hairpin intermediate state. Once the pre-fusion hairpin has formed, however, the epitope is likely obscured. For simplicity of illustration the conjoint CD4 and chemokine receptor binding to the gp120 protomer in the unshed state is omitted.

Supplementary Table 1. Cryo-EM data collection, refinement and validation statistics

	gp145 (EMDB-25022) (PDB: 7SC5)	gp145•3Fab (EMDB-25045) (PDB: 7SD3)	gp145•2Fab (EMDB-25025)	gp145•1Fab (EMDB-25024)
**Data collection and processing**				
Microscope	Titan Krios			
Voltage (kV)	300			
Detector	K2 Summit (Gatan)			
Pixel size (Å)	1.03			
Magnification	29,000			
Defocus range (μm)	-1.5 to -3.0			
Total dose (electrons/Å^2)	80			
Movie stacks(no.)	30404			
Initial particle images(no.)	3,039,896			
Particle images for final reconstruction (no.)	47,616	362,646	21,454	23,583
Symmetry imposed	C3	C1	C1	C1
Map resolution at 0.143 FSC threshold (Å)	3.9	3.7	8.2	8.8
**Refinement**				
Initial models used (PDB code)	5I8H	5I8H; 4XC3		
Map sharpening B factor (Å^2)	-110	-120		
Model composition				
Non-hydrogen atoms	14703	25256		
Protein residues	1770	3111		
Ligands	BMA:6 NAG:54	BMA:6 NAG:54		
B factors (Å^2)				
Protein	130.15	134.33		
Ligand	158.76	134.86		
R.m.s. deviations				
Bond lengths (Å)	0.008	0.011		
Bond angles (°)	1.248	1.475		
MolProbity score	2.11	2.77		
Clashscore	10.05	11.23		
Poor rotamers (%)	0.96	7.23		
Ramachandran plot (%)				
Favored/Allowed/Outliers	88.47/11.3/0.23	90.08/9.86/0.07		

SUPPLEMENTARY REFERENCES

1. Rantalainen, K. *et al.* HIV-1 Envelope and MPER Antibody Structures in Lipid Assemblies. *Cell Rep.* **31**, 107583 (2020).
2. Kwon, B., Lee, M., Waring, A. J. & Hong, M. Oligomeric Structure and Three-Dimensional Fold of the HIV gp41 Membrane-Proximal External Region and Transmembrane Domain in Phospholipid Bilayers. *J. Am. Chem. Soc.* **140**, 8246–8259 (2018).
3. Fu, Q. *et al.* Structure of the membrane proximal external region of HIV-1 envelope glycoprotein. *Proc. Natl. Acad. Sci. U. S. A.* **115**, E8892–E8899 (2018).
4. Irimia, A., Sarkar, A., Stanfield, R. L. & Wilson, I. A. Crystallographic Identification of Lipid as an Integral Component of the Epitope of HIV Broadly Neutralizing Antibody 4E10. *Immunity* **44**, 21–31 (2016).
5. Kong, R. *et al.* Fusion peptide of HIV-1 as a site of vulnerability to neutralizing antibody. *Science* **352**, 828–833 (2016).
6. Krebs, S. J. *et al.* Longitudinal Analysis Reveals Early Development of Three MPER-Directed Neutralizing Antibody Lineages from an HIV-1-Infected Individual. *Immunity* **50**, 677-691.e13 (2019).
