# Supplementary material for: Dynamic HIV-1 spike motion creates vulnerability for its membrane-bound tripod to antibody attack
Source: Nat Commun. 2022 Oct 27;13:6393. doi: 10.1038/s41467-022-34008-y (PMC9610346; doi:10.1038/s41467-022-34008-y)
Supplement: Supplementary file 3 — Description of Additional Supplementary Files [file 41467_2022_34008_MOESM3_ESM.pdf]

DESCRIPTION OF SUPPLEMENTARY MOVIES

Supplementary Movie 1. Motions of the HIV-1 Env ectodomain on the membrane surface. The movie was generated by morphing between 20 ectodomain orientations obtained by docking the ectodomain structure into EM maps obtained during image processing (Supplementary Fig. 3).

Supplementary Movie 2. Largest tilts of the HIV-1 Env ectodomain relative to the membrane surface. The movie was generated by morphing between the two ectodomain orientations showing the highest tilt angles.

Supplementary Movie 3. Coarse-grained molecular-dynamics simulation of membrane-embedded gp145. The movie shows 2 μs of one of the repeats. Note the spontaneous tilting of the ectodomain and the uncorrelated dynamics of the three MPER segments (MPER-N in green and MPER-C in magenta).

Supplementary Movie 4. Coarse-grained molecular-dynamics simulation of membrane-embedded gp145 with bound 4E10 Fab. The movie shows 0.9 μs of one of the repeats. Note that the ectodomain still adopts different tilts but that the average tilt is higher than in the unliganded gp145. Fab binding appears to stabilize the three MPER segments at different average angles (MPER-N in green and MPER-C in magenta).
