# Supplementary material for: Dynamic HIV-1 spike motion creates vulnerability for its membrane-bound tripod to antibody attack
Source: Nat Commun. 2022 Oct 27;13:6393. doi: 10.1038/s41467-022-34008-y (PMC9610346; doi:10.1038/s41467-022-34008-y)
Supplement: Supplementary file 8 — Reporting Summary [file 41467_2022_34008_MOESM8_ESM.pdf]

nature portfolio

Corresponding author(s): Ellis L. Reinherz, Thomas Walz

Last updated by author(s): Sep 6, 2022

# Reporting Summary

Nature Portfolio wishes to improve the reproducibility of the work that we publish. This form provides structure for consistency and transparency in reporting. For further information on Nature Portfolio policies, see our Editorial Policies and the Editorial Policy Checklist.

## Statistics

For all statistical analyses, confirm that the following items are present in the figure legend, table legend, main text, or Methods section.

| n/a | Confirmed | |
|---|---|---|
| ☐ | ☒ | The exact sample size (*n*) for each experimental group/condition, given as a discrete number and unit of measurement |
| ☐ | ☒ | A statement on whether measurements were taken from distinct samples or whether the same sample was measured repeatedly |
| ☒ | ☐ | The statistical test(s) used AND whether they are one- or two-sided<br>*Only common tests should be described solely by name; describe more complex techniques in the Methods section.* |
| ☒ | ☐ | A description of all covariates tested |
| ☒ | ☐ | A description of any assumptions or corrections, such as tests of normality and adjustment for multiple comparisons |
| ☐ | ☒ | A full description of the statistical parameters including central tendency (e.g. means) or other basic estimates (e.g. regression coefficient) AND variation (e.g. standard deviation) or associated estimates of uncertainty (e.g. confidence intervals) |
| ☒ | ☐ | For null hypothesis testing, the test statistic (e.g. *F*, *t*, *r*) with confidence intervals, effect sizes, degrees of freedom and *P* value noted<br>*Give P values as exact values whenever suitable.* |
| ☒ | ☐ | For Bayesian analysis, information on the choice of priors and Markov chain Monte Carlo settings |
| ☒ | ☐ | For hierarchical and complex designs, identification of the appropriate level for tests and full reporting of outcomes |
| ☒ | ☐ | Estimates of effect sizes (e.g. Cohen's *d*, Pearson's *r*), indicating how they were calculated |

*Our web collection on statistics for biologists contains articles on many of the points above.*

## Software and code

Policy information about availability of computer code

| | |
|---|---|
| Data collection | SerialEM 3.8 |
| Data analysis | RELION 3.0, MotionCor2, Gautomatch 0.56, CTFFIND4.1, cryoSPARC 2.47, cryoSPARC 2.48, Chimera 1.15, Coot 0.8.9, PHENIX 1.17.1, EMAN2, SPIDER, Martinize2, GROMACS 2020.6, MARTINI3, CHARMM-GUI, VMD v.1.9.4a12, Microsoft Excel 2016 |

For manuscripts utilizing custom algorithms or software that are central to the research but not yet described in published literature, software must be made available to editors and reviewers. We strongly encourage code deposition in a community repository (e.g. GitHub). See the Nature Portfolio guidelines for submitting code & software for further information.

## Data

Policy information about availability of data

All manuscripts must include a data availability statement. This statement should provide the following information, where applicable:

- Accession codes, unique identifiers, or web links for publicly available datasets
- A description of any restrictions on data availability
- For clinical datasets or third party data, please ensure that the statement adheres to our policy

The cryo-EM maps have been deposited in the Electron Microscopy Data Bank under accession codes EMD-25022 [https://www.ebi.ac.uk/pdbe/entry/emdb/ EMD-25022] (gp145), EMD-25024 [https://www.ebi.ac.uk/pdbe/entry/emdb/ EMD-25024] (gp145•1Fab), EMD-25025 [https://www.ebi.ac.uk/pdbe/entry/emdb/ EMD-25025] (gp145•2Fab) and EMD-25045 [https://www.ebi.ac.uk/pdbe/entry/emdb/EMD-25045] (gp145•3Fab). The atomic coordinates have been deposited in the PDB under accession codes PDB-7SC5 [https://doi.org/10.2210/pdb7SC5/pdb] (gp145) and PDB-7SD3 [https://doi.org/10.2210/pdb7SD3/pdb] (gp145•3Fab).

## Human research participants

Policy information about studies involving human research participants and Sex and Gender in Research.

| | |
|---|---|
| Reporting on sex and gender | N/A |
| Population characteristics | N/A |
| Recruitment | N/A |
| Ethics oversight | N/A |

Note that full information on the approval of the study protocol must also be provided in the manuscript.

# Field-specific reporting

Please select the one below that is the best fit for your research. If you are not sure, read the appropriate sections before making your selection.

☒ Life sciences ☐ Behavioural & social sciences ☐ Ecological, evolutionary & environmental sciences

For a reference copy of the document with all sections, see nature.com/documents/nr-reporting-summary-flat.pdf

# Life sciences study design

All studies must disclose on these points even when the disclosure is negative.

| | |
|---|---|
| Sample size | No statistical tests were used to predetermine sample size. |
| Data exclusions | Micrographs clearly suffering from astigmatism, image drift, ice contamination and/or hexagonal ice formation were excluded from the datasets. Particles in 2D classes showing no secondary structural features and in 3D classes showing unsatisfactory structural features were excluded from the final reconstructions. |
| Replication | Cryo-EM data were collected across five sessions. All attempts at replication were successful. |
| Randomization | Randomization is not applicable to our study, because no predetermined control and sample groups were used. |
| Blinding | Blinding is not applicable to our study, because no populations were preassigned to groups, so that blinding was not relevant. |

# Reporting for specific materials, systems and methods

We require information from authors about some types of materials, experimental systems and methods used in many studies. Here, indicate whether each material, system or method listed is relevant to your study. If you are not sure if a list item applies to your research, read the appropriate section before selecting a response.

## Materials & experimental systems

| n/a | Involved in the study | |
|---|---|---|
| ☐ | ☒ | Antibodies |
| ☐ | ☒ | Eukaryotic cell lines |
| ☒ | ☐ | Palaeontology and archaeology |
| ☒ | ☐ | Animals and other organisms |
| ☒ | ☐ | Clinical data |
| ☒ | ☐ | Dual use research of concern |

## Methods

| n/a | Involved in the study | |
|---|---|---|
| ☒ | ☐ | ChIP-seq |
| ☒ | ☐ | Flow cytometry |
| ☒ | ☐ | MRI-based neuroimaging |

# Antibodies

| | |
|---|---|
| Antibodies used | Fab PG9 (Kwong et al. (2009) Cell Host Microbe 6: 292-294)<br>Fab 4E10 (Chen & Dierich (1996) Immunol Lett 52: 153-156)<br>cDNAs for PG9 and 4E10 were obtained from Dr. Peter Kwong at the Vaccine Research Center of the National Institute of Allergy and Infectious Diseases, NIH, and were used for antibody expression, purification and Fab production. Fabs were used undiluted to form complexes with gp145. |
| Validation | The validation of each primary antibody is described in their associated publication, as referenced in the above section. |

# Eukaryotic cell lines

Policy information about cell lines and Sex and Gender in Research

| | |
|---|---|
| Cell line source(s) | Gibco Sf9 cells (ThermoFisher)<br>FreeStyle 293-F cells (ThermoFischer). |
| Authentication | We did not further authenticate the cell lines. |
| Mycoplasma contamination | We did not test for mycoplasma. |
| Commonly misidentified lines (See ICLAC register) | No commonly misidentified lines were used in our study. |
